# The optimal spatial noise for continuous flash suppression masking is pink

**DOI:** 10.1038/s41598-020-63888-7

**Published:** 2020-04-24

**Authors:** Jan Drewes, Weina Zhu, David Melcher

**Affiliations:** 1 Physics of Cognition Group (PHKP), Chemnitz University of Technology, 09126 Chemnitz, Germany; 2 School of Information Science, Yunnan University, 650091 Kunming, China; 3 Center for Mind/Brain Sciences (CIMeC), University of Trento, 38068 Rovereto, Italy; 4 Department of Psychology and Cognitive Science, University of Trento, 38068 Rovereto, Italy

**Keywords:** Visual system, Consciousness, Perception

## Abstract

A basic question in cognitive neuroscience is how sensory stimuli are processed within and outside of conscious awareness. In the past decade, CFS has become the most popular tool for investigating unconscious visual processing, although the exact nature of some of the underlying mechanisms remains unclear. Here, we investigate which kind of random noise is optimal for CFS masking, and whether the addition of visible edges to noise patterns affects suppression duration. We tested noise patterns of various density as well as composite patterns with added edges, and classic Mondrian masks as well as phase scrambled (edgeless) Mondrian masks for comparison. We find that spatial pink noise (1/F noise) achieved the longest suppression of the tested random noises, however classic Mondrian masks are still significantly more effective in terms of suppression duration. Further analysis reveals that global contrast and general spectral similarity between target and mask cannot account for this difference in effectiveness.

## Introduction

A basic question in cognitive neuroscience is how sensory stimuli are processed within and outside of conscious awareness. One of the main sources of evidence that stimuli that do not reach conscious awareness - and are thus subjectively “invisible” - are still processed to some degree by the visual system results from studies employing a technique known as Continuous Flash Suppression (CFS)^1^. Since its conception, originally in the context of afterimages, it has been used to present evidence for the unconscious (or unaware) processing of a number of low-level visual features, such as orientation^1,2^, motion^3^ or spatial information^4^, as well as the unconscious binding of low-level visual features based on Gestalt grouping cues, such as good continuation and proximity^5^. Even some effects commonly attributed to “high-level” visual processing stages have been reported to be occur without the subject being aware of the percept, for example in face inversion^6–8^, facial expressions^9,10^, semantic information processing^6,11,12^ and information integration^13–15^. Probably due to its power and versatility, in recent years CFS has become widely used to investigate visual processing outside of (subjective) conscious awareness. Still, the exact nature of the underlying neuronal mechanisms remains poorly understood^16–20^.

In a typical CFS paradigm, a series of salient masks (e.g. colorful and contour-rich) are continuously flashed to only one eye, causing a static (e.g. low contrast) image presented only to the other eye to be suppressed from perceptual awareness throughout a period of time significantly longer than what would be expected from binocular rivalry alone^1,21^. In many of the recent studies, the suppression masks were designed from overlapping rectangles of different color or luminance, the result appearing similar to the work of the artist Pieter Cornelis Mondriaan (Mondrian). Unfortunately, a large number of studies utilizing CFS do not specify in sufficient (or even any) detail how their respective masks were designed^19,20^. In our previous research, we evaluated the CFS effectiveness of different mask types, given that the features of the mask itself might provide additional insight into the nature of suppression from conscious awareness. We found that the CFS effectiveness was influenced by both temporal and spatial factors^19,20^. In terms of spatial features, we investigated mask density (the average size and number of patches per mask) and, in consequence, the spectral density, including both the cardinal and oblique directions of the spectrum^19^. We found that CFS effectiveness (i.e., suppression duration) was negatively affected when reducing the number of edges in the masks.

Recently, the methodology of CFS and in particular the type of mask used to achieve suppression has been the subject of some attention, with studies addressing both temporal^19,20,22^ and spatial aspects^19,23^ of CFS masking. However, it is not clear from those results whether spatial density in general, or more specifically the number of edges, influenced the effectiveness of the masking. To investigate this question, we created a set of masks that vary in spatial density, but do not contain edges as would be found in Mondrian-style masks. These are similar to random noise stimuli and offered themselves for this purpose, as they are easy to control in terms of spectral properties. We therefore specifically aim to avoid the edge detection mechanisms in the visual system. Subsequently, we examine if a re-introduction of Mondrian-like edge patterns would lead to an enhanced suppression duration, and if and how much suppression effectiveness is reduced when removing the edges from the Mondrian masks.

## Methods

### Apparatus

We presented visual stimulation on a 21 inch Mitsubishi CRT monitor (1024 × 768 pixel resolution, 160 Hz frame rate). Subjects’ heads were stabilized by a chin-and-head rest, while viewing the presentation through a mirror stereoscope. The physical viewing distance was 57 cm, while the optical distance was increased by approx. 9.5 cm due to the detour through the mirror stereoscope. The spatial distance between the left and right presentation areas as well as the angle of the mirrors were adjusted for each observer to achieve good fusion of the display. Visual stimuli were presented in MATLAB (TheMathWorks, Inc., 2012) using the Psychophysics Toolbox^24^.

### Stimuli and masks

#### Target stimuli

**Figure 1:**
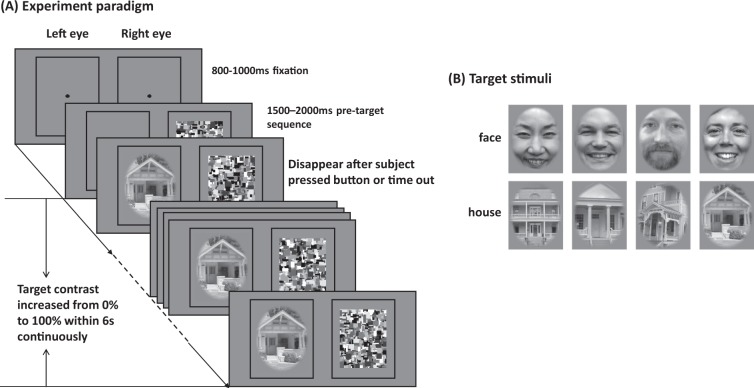
(**A**) Experimental paradigm (breaking-CFS). The contrast of the stimulus images increased continuously from 0% to 100% over a period of 6 s. Subjects were instructed to press the space button as quickly as possible when they saw an image. By this design, the measured breakthrough contrast was equivalent to the response time or duration of suppression. (**B**) Sample of target stimuli: faces and houses. In order to comply with copyright requirements, the original face images were replaced with stand-ins that were preprocessed in an identical fashion and match the visual appearance of the actual stimuli. All images copyright of the authors where applicable.

We used photographic grayscale face and house images (80 each) as target stimuli in this study. Images were preprocessed identically to^19,20^. Images measured 6.8 by 8.5 degree visual field. Examples of the resulting stimulus images are shown in Fig. 1B.

#### Mask stimuli

**Figure 2:**
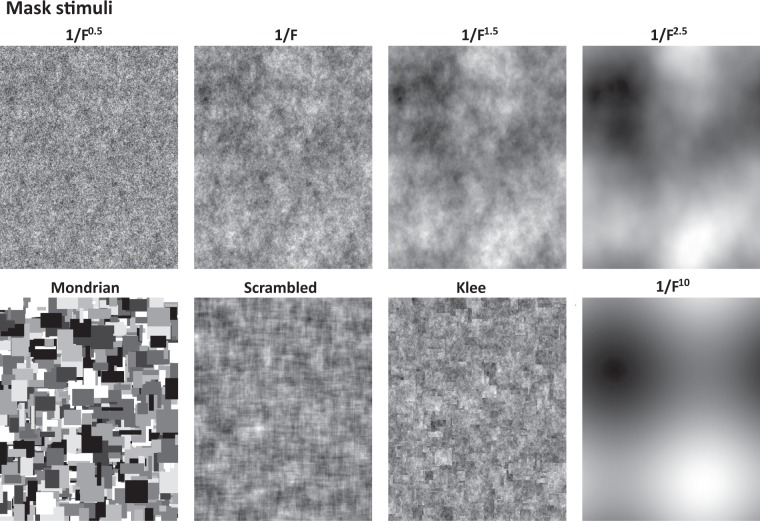
Sample of Mask stimuli: Five random noise masks, normal Mondrian masks, phase-scrambled Mondrian masks and noise composite masks (Klee-masks).

**Figure 3:**
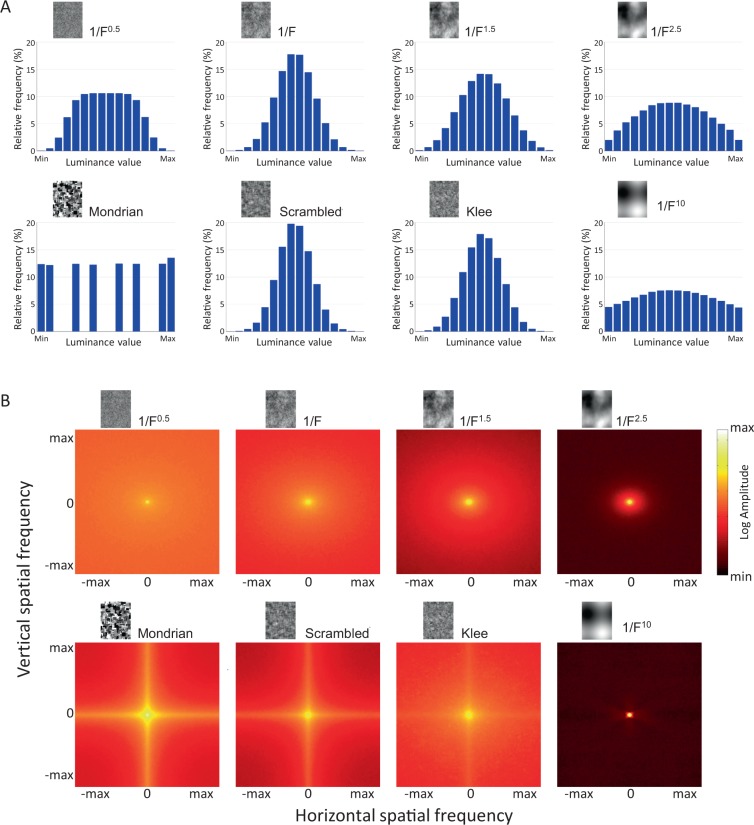
Mask statistics. (**A**) Luminance value distribution across different mask types (pixel analysis). (**B**) Mean Fourier amplitude spectra of mask types, corresponding to (**A**).

In this study, we evaluated the CFS effectiveness of eight different mask types. Five of them were random spatial noise masks that were designed to vary in their spectral properties. Another three types of masks include normal Mondrian masks, phase-scrambled Mondrian masks and noise composite masks (“Klee”-masks, named for the similarity to a series of artworks by the artist Paul Klee). An overview of all used mask types can be seen in Fig. 2. For each type, 200 individual masks were generated. Each individual mask was generated with a Michelson contrast of 1, to allow for the highest possible contrast within each mask without compromising the desired spectral properties. On average, all masks had a mean luminance of 50%. General mask statistics can be seen in Fig. 3.

#### Random noise masks

We created uniform white noise and modulated the amplitude spectrum to follow a characteristic of 1/F^n^, with n = [0.5, 1, 1.5, 2.5, 10]. In this way, we could control the density of the mask without introducing edges^19^, while maintaining a naturalistic frequency distribution pattern. In particular, pink noise (i.e., n = 1) is often considered to be an “optimal” stimulus for the visual system, as it resembles the average frequency distribution of natural scenes^25,26^.

#### Normal mondrian masks

Mondrian masks typically involve a randomization process that combines different, overlapping rectangles. The normal Mondrian masks used in this study (see also^20^) were created by a computer algorithm introduced previously^19^, which are visually similar to CFS masks used in other studies.

#### Scrambled mondrian masks

The Scrambled Mondrian masks were generated from the above grayscale Mondrian masks by means of phase scrambling in the Fourier domain. Through this, scrambled Mondrian masks have very similar spatial frequency energy distribution as normal Mondrian masks, but without coherent edges.

#### Klee masks

Klee masks were created in an attempt to combine the edge patterns of Mondrian masks with the frequency characteristics of pink noise, chosen due to its specific frequency characteristics (see “Random Noise Masks”). Procedurally, Klee masks were designed from normal Mondrian masks by replacing the pixels of each gray level with the corresponding pixels from pink (1/F) noise masks of eight different random seeds (one for each gray level). Pink noise patterns differ from white noise through local spatial correlations due to the higher amount of low spatial frequencies. These local correlations are broken along the borders between random seeds; the result are patterns of pink noise that incorporate (second-order) edges between different noise patches, of the same count, location and orientation as their Mondrian equivalents.

### Experimental paradigm

In this study, a breaking-CFS paradigm was used, in which the masks were flashed to the dominant eye at predefined frequencies, while the target stimulus image was presented to the other eye, as illustrated in Fig. 1(A). Recently, Ding *et al*. suggested that eye dominance should be determined using a pretest of the same task that will be used in the experiment^27^. However, in order to be consistent with our previous studies, we continued to use the ABC test^28,29^ to determine the dominant eye. There were two static frames (10.2° ×12.6°) surrounding the outer border of target stimuli and masks presented on the two sides of the screen, such that both frames were visible only to their respective corresponding eye. In order to achieve good fusion between both frames, prior to the first trial of each block, the mirror setup as well as the distance between the two surrounding frames were adjusted individually for each subject. Each trial started with a central fixation cross extending 1°, shown for a random period of 800–1000 ms. The presentation size of the masks was 8.5° × 10.6°. The masks were flashed for a random period of 1500–2000 ms before the target stimulus began to ramp up to the other eye. The contrast of the stimulus image increased from 0% to 100% linearly in 6 s (around 0.1% per display refresh with 8 bit precision), while the repetitive mask display to the dominant eye continued. Participants were instructed to press the space button as quickly as possible when they saw any part of the target image, but not before they could see the target (breaking CFS, b-CFS). Subjects were informed that they would also experience trials without target (catch trials), on which they were not to give a response. After subjects pressed the button, or after the trial timed out (6 s), images disappeared.

Every subject completed 8 blocks with one mask type per block. The order of mask types was randomized across subjects. In each block there were 78 face trials, 78 house trials and 24 empty (no target stimulus) catch trials, which were balanced across six masking frequencies (3, 5, 7, 10, 13 and 16 Hz, similar to our approach in previous studies^19,20^). All of the images were presented in random order, and only once per block for each subject. All frequencies occurred equally often in each block. A total of 180 trials were collected in each block, with 26 target-present trials and 4 catch trials per frequency, resulting in a total of 1440 trials per subject. Subjects ran these blocks as three sessions on three separate days.

### Subjects

Nineteen subjects participated in the experiment (15 female, aged 19–35: mean = 23.3, SD = 4.3). All participants were students or postdoctoral fellows recruited from the University of Trento and were paid for their participation. The participants reported normal/corrected-to-normal vision and were naive to the purpose of the experiment.

### Statistical analysis

The mean of the breakthrough contrast (determined as the percentage of contrast at the time when the subject pressed the button) of each mask type and each masking frequency was computed per subject on log-transformed data. For comparisons, as indicated, the break-through contrast was analyzed by an ANOVA design for repeated measures with the masking frequency (6 different frequencies), mask type (8 levels) and stimulus type (faces vs. houses) as within-subject factors. Greenhouse–Geisser adjustments to the degrees of freedom were applied when appropriate and are reported in subscript.

### Ethics statement

The experiment was approved by the ethics committee of Trento University, and performed according to the principles expressed in the Declaration of Helsinki. Written informed consent was obtained from all participants.

## Results

We found significant main effects of the type of mask: F (7_2.856_,126_51.41_) = 20.06, p < 0.001. The classic Mondrian masks appeared to be the most effective type of masks, causing the highest overall break-through contrast/longest suppression duration (mean of 40.5% contrast, equaling 2.43 s). It appears that among the random noises, pink noise (1/F) had the highest masking effectiveness (1/F^0.5^ = 1.39 s, 1/F^1^ = 1.63 s, 1/F^1.5^ = 1.59 s, 1/F^2.5^ = 1.16 s and 1/F^10^ = 0.96 s respectively). There was, however, no significant difference between pink noise, scrambled Mondrians and Klee masks (pink noise = 1.63 s, scrambled Mondrians = 1.71 s and Klee masks = 1.69 s respectively) (see Fig. 5A). The statistical results of the pairwise comparisons in mean masking suppression contrast between types of masks can be found in Table 1.

**Figure 4:**
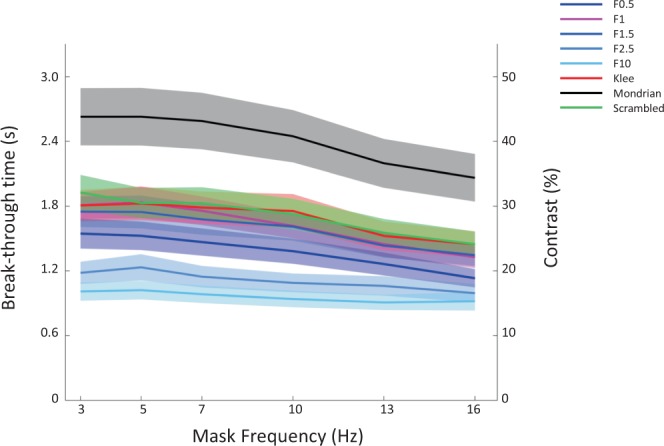
Main results. Break-through contrast/time over temporal frequencies; colors indicate mask types. Mean and s.e.m. across subjects.

**Figure 5:**
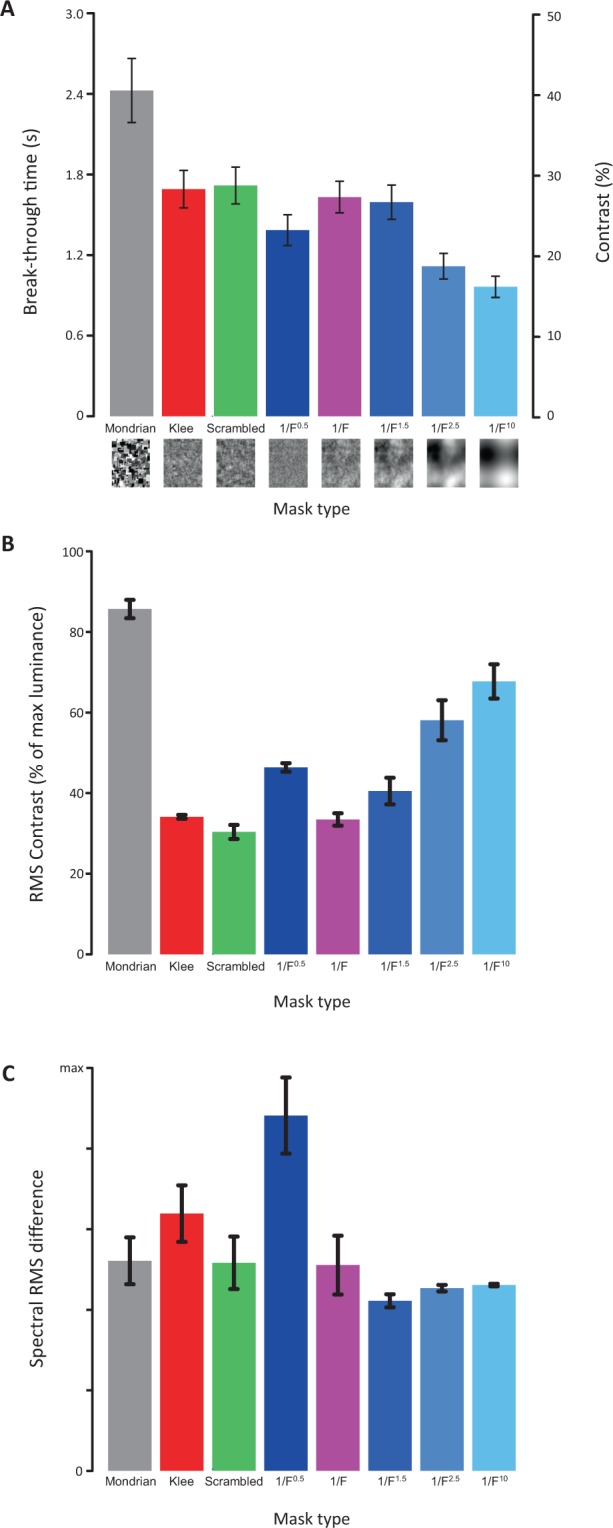
Results per type of mask. (**A**) Break-through contrast and time over all mask types (mean and 1 s.e.m across subjects). (**B**) RMS within-mask contrast (mean and sd across masks). (**C**) Spectral RMS difference between masks and targets (mean and sd across mask/target pairs).

**Table 1:** Pairwise statistical comparisons of mean suppression contrast between mask types.

	Mondrian	Scrambled	Klee	F^0.5^	F^1^(Pink)	F^1.5^	F^2.5^	F^10^
Mondrian								
Scrambled	**0.039**							
Klee	**0.019**	1.000						
F^0.5^	**0.000**	**0.044**	0.200					
F^1^(Pink)	**0.019**	1.000	1.000	**0.039**				
F^1.5^	**0.019**	1.000	1.000	0.532	1.000			
F^2.5^	**0.001**	**0.019**	**0.028**	**0.000**	**0.000**	**0.000**		
F^10^	**0.000**	**0.019**	**0.019**	0.272	**0.000**	**0.019**	0.216	

Bonferroni-Holm corrected p values, significant values are emphasized in bold (α < 0.05).

All mask types appeared to follow the same overall temporal trend, with the lowest break-through contrast around 16 Hz (see Fig. 4). In other words, masking was more effective for low temporal frequencies, in the theta range, consistent with previous findings^20^. The main effect of temporal masking frequency was also significant: F(5_1.521_,90_27.39_) = 40.093, p < 0.001. For the Normal Mondrian mask, replicating previous results^19,20^, the break-through contrast differed significantly between temporal masking frequencies (F(5_1.674_,90_30.138_) = 10.482, p < 0.001) and the most effective temporal frequency was in the theta band. The same trend held also for the other mask types (see Table 2). There was significant interaction between temporal masking frequency and type of mask(F(35_5.678_,630_102.21_) = 3.678 p = 0.003) which is also consistent with our previous results^19,20^.

**Table 2:** Dependency of masking effectiveness on mask temporal frequency.

Type of mask	F-statistic	p
Mondrian	F(5_1.674_,90_30.138_)	<**0.001**
Scrambled Mondrian	F(5_2.596_,90_56.735_)	<**0.001**
Klee	F(5_2.337_,90_42.067_)	<**0.001**
Noise 1/F^0.5^	F(5_1.848_,90_33.265_)	<**0.001**
Noise 1/F^1^	F(5_2.603_,90_46.852_)	<**0.001**
Noise 1/F^1.5^	F(5_2.152_,90_38.729_)	<**0.001**
Noise 1/F^2.5^	(F(5_2.150_,90_38.702_)	<**0.001**
Noise 1/F^10^	F(5_2.600_,90_46.809_)	=**0.001**

Repeated measures ANOVA, Greenhouse-Geisser corrected F-statistic and p-values (significant values emphasized in bold, α < 0.05).

The break-through contrast differed significantly between the different target stimuli (faces and houses): F(1_1.000_,18_18.00_) = 24.97 p < 0.001 (faces = 1.46 s; houses = 1.67 s), again replicating the results reported previously^19,20^. Furthermore, subjects responded only rarely on catch trials (Avg = 3.7%, SD = 4.7%, Max = 18.8%) indicating good compliance with the instructions given.

**Figure 6:**
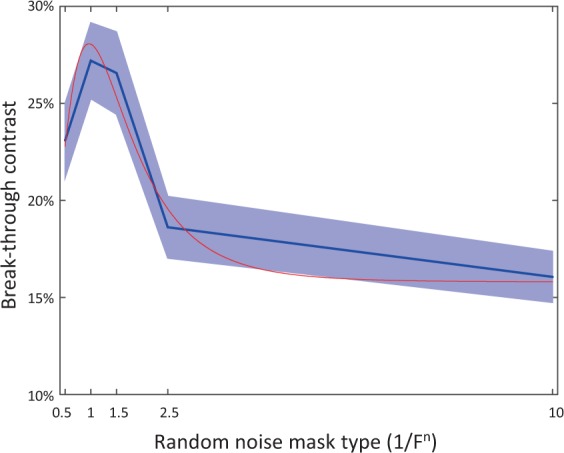
Function fitting results. Break-through contrast (percent) over random noise mask types, mean and s.e.m. across subjects. Red line indicates best-fit skewed Gaussian.

In this experiment, we hypothesized that the masking effectiveness of pink noise with edges (Klee masks) would be better than pink noise itself. It turned out that the Klee mask did not achieve significantly more masking effectiveness than pink noise. This result led us to investigate whether pink noise is in fact the most effectual noise to design Klee-style masks, or whether a different fall-off characteristic would be optimal. In order to test this, a skewed, raised Gaussian function was fitted to the mean breakthrough contrast of all the different random noise mask types (see Fig. 6). The analysis of the fitted curve indicates the maximum to be at n = 1.01 (mean value of bootstraps across subjects, N = 10k, 95% confidence from 0.76 to 1.182), indicating that pink (1/F^1^) is indeed the optimum frequency fall-off for generating CFS masks. This result is aligned with previous findings measuring the average duration of dominance in binocular rivalry, in which pink noise was found to be the preferred noise stimulus to achieve the longest average dominance periods when compared to other noise spectra^30^. However, aside from using classic binocular rivalry as a methodology, that previous study differed from the current one in that it compared noise stimuli against other noise stimuli, rather than using natural scene targets as in the current study. Also, in our previous study^19^, we found that white noise was a weak CFS masking stimulus, whereas in their binocular rivalry study Baker and Graf present data that shows white noise to be relatively stronger than 1/F^2^ and similar to 1/F^1.5^ noise, which would place it very close to the suppression duration of the optimally effective pink noise in the current study.

### Contrast analysis

An alternative explanation for the difference in suppression effectiveness of the different mask types might be the within-mask contrast, as suppression duration in conventional binocular rivalry is known to depend heavily on contrast^31^. While all masks were scaled to their respective maximum contrast within the limits of the display system (Michelson contrast of 1, see “Stimuli and Masks”), RMS contrast was allowed to differ between mask types. Figure 5B shows the average RMS contrast for each mask type. Indeed, Mondrian masks have the highest contrast, which is explained by their design (each luminance occurs equally often, leading to a white luminance histogram – see Fig. 3A). However, for the remaining mask types, RMS contrast and suppression effectiveness do not correspond well, and in the case of the 1/F^n^ noises, even exhibits a negative correlation, with Pink noise masks having the narrowest luminance histogram and therefore the lowest average RMS contrast. Therefore, while contrast may certainly be a contributing factor, it cannot serve as the sole explanation of the varying mask effectiveness of the different mask types.

### Spectral similarity analysis

The stimuli used in this study were photographs of human faces and houses, with a general spectral frequency characteristic similar to most natural scenes. Similarity between mask and target has been shown to be a factor in both binocular rivalry^32^ as well as CFS^33^. It would therefore be conceivable that some of the mask stimuli used in this study may be more similar in their spectral profile to the target stimuli than others, which may again explain the differences in masking efficiency. We computed the spectral difference relative to the targets for each mask type by subtracting the spectra of each mask from the spectra of each stimulus image and then computing the RMS difference. Prior to this, each spectrum was normalized to a total sum of 1 to minimize effects of global contrast. The result can be seen in Fig. 5C, with larger bars indicating less similarity. From this, the highest degree of similarity is found with the 1/F^1.5^ masks, and the lowest similarity with the 1/F^0.5^ masks. Pink noise, scrambled Mondrians as well as Mondrians result in virtually identical similarity measures (with only the tiniest of advantages for the Pink noise masks), while Klee masks rank second to last. This is at odds with the pattern found in our experimental results. General similarity of the amplitude spectrum can therefore not explain the ordering of the masking effectiveness found in this study. Overall, the findings with the RMS contrast and spectral difference analyses indicate that while binocular rivalry and CFS certainly involve many shared mechanisms and many properties are comparable, not all findings from binocular rivalry can be applied directly to CFS.

### On the contribution of temporal frequency signatures to masking effectiveness

**Figure 7:**
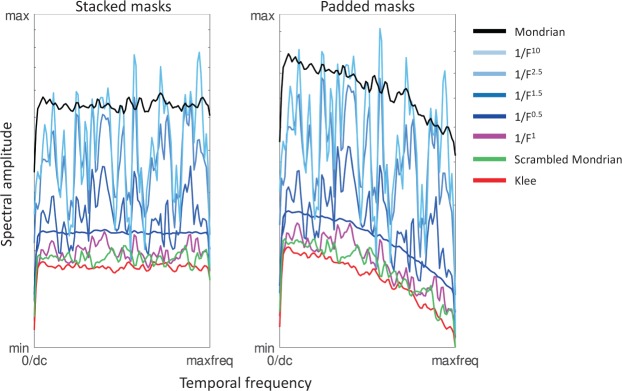
Temporal frequency signatures of different types of masks. Left panel: directly stacked masks, resulting in white noise spectra. Right panel: masks padded 20x to simulate the 5 Hz masking condition on a 100 Hz display system.

On the stimulus level (i. e., the display system), it has repeatedly been shown that CFS is generally most effective with a mask replacement frequency in the theta band, around 6 Hz^1,19,20^. On the neural level, it has been shown that very low frequencies (close to 1 Hz) result in the most effective masking^22^. The reason for the apparent difference however is simply the point of view. One may see the masks presented in any CFS trial as a three-dimensional pixel structure: all the successive spatial mask structures stacked on top of each other, allowing us to trace any given pixel through the third dimension (time). As the masks were all created independently in space (2D), the temporal successors and predecessors of each pixel will be uncorrelated with each other, resulting in a temporal spectral signature corresponding to white noise. This spectral signature is independent of the spatial spectral slope of the masks and the corresponding luminance histograms, except for a general amplitude effect: masks with higher spatial luminance contrast also result in increased temporal luminance contrast and thus higher spectral amplitude (see Fig. 7). However, this temporal white noise characteristic does not immediately apply to the way in which masks are actually presented to the observer during a typical CFS experiment. Individual masks are not updated for each new frame of the display system (100 Hz in this study), but are repeated for multiple successive frames to achieve the desired mask presentation rate (e. g. 5 Hz, resulting in 20 repetitions of each mask). The stack of masks is therefore “padded” with multiple repetitions of each mask. From the perspective of the visual system, this model thus includes periods of static mask display with regular but relatively sparse changes of the displayed mask. Fourier transform along the temporal axis of the padded mask stacks reveals a temporal frequency spectrum that emphasizes the lower temporal frequencies and attenuates the higher temporal frequencies (see Fig. 7), consistent with the work of Han *et al*.^22^. This is, however, again independent of the spatial frequency spectrum of the masks and can therefore not explain the differences in masking strength of the different types of masks examined in this study.

## Discussion

In this study, we employed a breaking-CFS paradigm in order to evaluate the CFS effectiveness of a set of 8 different suppression masks, including the popular Mondrian type of mask, a scrambled version of the Mondrian masks, noise-composite Klee masks, and a series of random noise masks with varying frequency fall-off characteristics. The main goal of the experiment was to test whether these different types of noise masks, with and without added line structure, differed in their ability to suppress the face/house image from visual awareness. The highest suppression effectiveness was found with the Mondrian masks, which differed significantly from all other mask types tested here.

In our previous study^19^, we postulated that edges might be a critical feature contributing to the effectiveness of the Mondrian masks. The importance of edges in visual masking (in dichoptic masking as well as binocular rivalry) has been shown before, although the addition of phase-aligned structures to the masks did not always increase masking strength^34,35^. Here, indeed we find that, when removing those edges by phase-scrambling the mask images, the resulting scrambled Mondrian masks appear to be significantly less effective than our original Mondrians. We attempted to add edges back into random noise masks in order to see if their suppression effectiveness could thereby be raised to the level of the Mondrians. Instead, the added edge structure did not increase mask effectiveness. Through direct measurements, as well as the evaluation of a function fitted across the results of all the tested noise masks, we were able to determine that the well-known frequency fall-off characteristics of pink noise (1/F^1.0^) was an optimal design parameter for random noise masks used in CFS. It has previously been shown that naturalistic spectra, specifically pink noise, are a preferred stimulus in binocular rivalry^30^; however, the findings from binocular rivalry do not always apply equally to continuous flash suppression (see the contrast analysis of the results section), which points to the fact that CFS is not just a boosted form of binocular rivalry, but differs in its characteristics and may therefore involve different aspects of the visual system – even though a large degree of overlap may be assumed. The main procedural difference between CFS and conventional binocular rivalry is in the mask dynamics, which are on a rather fast timescale compared to the typical dominance durations found in binocular rivalry^31,36^. It might be possible that this prevents the expression of certain slower-paced characteristics of binocular rivalry altogether, or resets their time course with the appearance of each new mask, preventing these characteristics from reaching full strength.

Pink noise resembles the frequency statistics of natural scenes^25,26,37,38^. It is widely believed that the human visual system is specifically adapted to process visual content of this make-up. It may be because of this that pink noise is also an optimal random noise mask for CFS, as it may bind visual cognitive resources in a way that is optimal to suppress the target stimulus.

The fact that pink noise is an optimal noise stimulus for CFS supports our decision to construct our Klee-type masks from the spatial patterns (edges) of the Mondrian masks, but replacing the plain grey level rectangles by patches of pink noise. In doing so, we had hoped to combine the edge pattern of the Mondrian masks with the otherwise optimal, but edge-less qualities of pink noise. Our hypothesis was that the Klee masks would perform (i.e., suppress the target) noticeably better than pink noise alone, and perhaps even rival the Mondrian patterns themselves. Contrary to our expectations, the Klee masks achieved little or no advantage over the pink noise masks, not reaching statistical significance. Klee masks, by means of their design, have the pixel patterns of pink noise with added edges of the same frequency, location and orientation as their Mondrian equivalents. However, the edges do not separate uniform luminance patches, but noise patches that differ in their respective random seed. While the edges are visible to the observer and result in emphasized vertical and horizontal orientations in the amplitude spectrum (see Methods, Fig. 3), the edges do not seem to improve the suppression effectiveness in a meaningful way.

Subjectively, the edges did also create corners and some proto-shapes, but evoked less of an impression of figure-ground segmentation and the creation of unique surfaces or objects. Scene segmentation at the level of surfaces has been suggested to be a key stage in visual processing^39,40^. In addition, the nature of the edges in our Klee masks present a possible explanation for this finding: the edges between two noise patches were not uniform in contrast, and to the visual system were perhaps closer to second-order effects rather than first-order edges, as found in conventional Mondrian masks. Also, since the edges arise from sudden changes in the underlying random seed, they are more obvious in the lower spatial frequencies, where the neighborhood correlations between pixels are stronger than in the higher spatial frequencies. At the location of a Klee-style edge, these correlations are suddenly interrupted. At higher spatial frequencies, these neighborhood correlations are reduced, and, nearing the Nyquist frequency, these local correlations even approach zero - therefore rendering transitions between random seeds (and therefore the presence of an edge) undetectable.

Regarding the question whether spatial density or rather the number of edges most affected suppression effectiveness^19^, it appears that density was a key factor in determining the effectiveness of the random noise masks used in this study. However, the presence of (first-order) edges or surfaces apparently matters strongly as well, as otherwise the significant decline in suppression effectiveness between Mondrians and their scrambled equivalents would remain unexplained.

Another potential explanation for the lack of improvement in the Klee masks, as well as the loss of suppression effectiveness when removing the edges from the Mondrian masks by means of phase scrambling, might have been the fact that – contrary to our hypothesis – the edges might not be the most important factor separating the Mondrian masks from the other mask types evaluated in this study. As we can see from Fig. 3, all of the noise stimuli, including the Klee masks and the scrambled Mondrians, have luminance distributions resembling Gaussian density functions. Therefore, the middle gray levels are most frequent, and the further away a luminance value is from the 50% mean, the less frequent it occurs, reducing the average contrast between gray levels. The Mondrian masks on the other hand are in our comparison the only masks with a white (flat) luminance histogram – they were constructed from equally many patches of each of the 8 gray levels, i.e. there are as many fully white and fully black pixels as there are pixels of each of the intermediate levels. This causes the Mondrian masks to exhibit higher average RMS contrast within each mask, as each luminance level occurs equally often and medium luminance values are therefore not favored. For the scrambled Mondrians, this feature is lost (see Fig. 3). Contrast is known to be a major factor in binocular rivalry^31^; still, while the comparison of RMS contrast for each mask type with the experimental results did confirm the higher contrast of the Mondrian masks, it could not confirm this as the general underlying reason for the differences in suppression duration. Similarity between targets and masks, which has been shown to be a factor in binocular rivalry^32^ as well as CFS^33^, could also not be confirmed as an explanation for the experimental results.

The concentration of Fourier energy on the horizontal and vertical axis of the spectrum might be a further contributing factor, as it appears to be strongest with the Mondrians (by design), and somewhat less with the scrambled Mondrians, even less with the Klee masks and ultimately absent with the pink noise masks (and all other random noise masks used here). The concentration of Fourier energy on the cardinal axis may easily be removed by choosing a different mask design; However, as the masks used in this study were not designed to systematically control for this factor, we cannot in sufficient rigor address this particular question directly. We do note however that this factor apparently did not have a significant impact on the results of the spectral similarity analysis.

We would conclude that spectral density and the presence of (first-order) edges are two important components in the design of effective CFS suppression masks. For optimal suppression in most cases, Mondrians still seem to be the masks of choice, at least when constructed as recommended previously^20^. In settings where random noises are preferable, a frequency characteristic of 1/F^1^ should generally be most effective. It remains an open question whether these factors reflect the ability of these properties to attract visual processing, or rather to engage it more fully, leading to greater competition and suppression of the weaker stimulus competing for awareness.

## Data Availability

All data will be made publically available at a suitable, open-access repository at time of publication.

## References

[1] Tsuchiya N, Koch C (2005). Continuous flash suppression reduces negative afterimages. Nat Neurosci.

[2] Montaser-Kouhsari L, Moradi F, Zandvakili A, Esteky H (2004). Orientation-selective adaptation during motion-induced blindness. Perception.

[3] Kaunitz L, Fracasso A, Melcher D (2011). Unseen complex motion is modulated by attention and generates a visible aftereffect. J Vis.

[4] van Boxtel JJ, Tsuchiya N, Koch C (2010). Opposing effects of attention and consciousness on afterimages. Proc Natl Acad Sci USA.

[5] Mitroff SR, Scholl BJ (2005). Forming and updating object representations without awareness: evidence from motion-induced blindness. Vision Res.

[6] Jiang Y, Costello P, He S (2007). Processing of invisible stimuli: advantage of upright faces and recognizable words in overcoming interocular suppression. Psychol Sci.

[7] Stein T, Hebart MN, Sterzer P (2011). Breaking Continuous Flash Suppression: A New Measure of Unconscious Processing during Interocular Suppression?. Front Hum Neurosci.

[8] Zhou G, Zhang L, Liu J, Yang J, Qu Z (2010). Specificity of face processing without awareness. Conscious Cogn.

[9] Jiang Y (2009). Dynamics of processing invisible faces in the brain: automatic neural encoding of facial expression information. Neuroimage.

[10] Smith ML (2012). Rapid Processing of Emotional Expressions without Conscious Awareness. Cereb Cortex.

[11] Costello P, Jiang Y, Baartman B, McGlennen K, He S (2009). Semantic and subword priming during binocular suppression. Conscious Cogn.

[12] Kang MS, Blake R, Woodman GF (2011). Semantic analysis does not occur in the absence of awareness induced by interocular suppression. J Neurosci.

[13] Lin Z, He S (2009). Seeing the invisible: the scope and limits of unconscious processing in binocular rivalry. Prog Neurobiol.

[14] Lin Z, Murray SO (2014). Unconscious processing of an abstract concept. Psychol Sci.

[15] Mudrik L, Breska A, Lamy D, Deouell LY (2011). Integration without awareness: expanding the limits of unconscious processing. Psychol Sci.

[16] Moors P, Hesselmann G, Wagemans J, van Ee R (2017). Continuous Flash Suppression: Stimulus Fractionation rather than Integration. Trends Cogn Sci.

[17] Yang E, Brascamp J, Kang MS, Blake R (2014). On the use of continuous flash suppression for the study of visual processing outside of awareness. Front Psychol.

[18] Sterzer P, Stein T, Ludwig K, Rothkirch M, Hesselmann G (2014). Neural processing of visual information under interocular suppression: a critical review. Front Psychol.

[19] Drewes J, Zhu W, Melcher D (2018). The edge of awareness: Mask spatial density, but not color, determines optimal temporal frequency for continuous flash suppression. J Vis.

[20] Zhu, W., Drewes, J. & Melcher, D. Time for Awareness: The Influence of Temporal Properties of the Mask on Continuous Flash Suppression Effectiveness. *PLoS One* **11**, e0159206, 10.1371/journal.pone.0159206 PONE-D-16-03644 [pii] (2016). 10.1371/journal.pone.0159206 PMC4945020 27416317

[21] Tsuchiya N, Koch C, Gilroy LA, Blake R (2006). Depth of interocular suppression associated with continuous flash suppression, flash suppression, and binocular rivalry. J Vis.

[22] Han S, Lunghi C, Alais D (2016). The temporal frequency tuning of continuous flash suppression reveals peak suppression at very low frequencies. Sci Rep.

[23] Han S, Alais D, Blake R (2018). Battle of the Mondrians: Investigating the Role of Unpredictability in Continuous Flash Suppression. Iperception.

[24] Brainard DH (1997). The Psychophysics Toolbox. Spatial Vision.

[25] Wichmann FA, Drewes J, Rosas P, Gegenfurtner KR (2010). Animal detection in natural scenes: critical features revisited. J Vis.

[26] Torralba A, Oliva A (2003). Statistics of natural image categories. Network: Computation in Neural Systems.

[27] Ding Y, Naber M, Gayet S, Van der Stigchel S, Paffen CLE (2018). Assessing the generalizability of eye dominance across binocular rivalry, onset rivalry, and continuous flash suppression. J Vis.

[28] Miles WR (1929). Ocular dominance demonstrated by unconscious sighting. Journal of Experimental Psychology.

[29] Miles WR (1930). Ocular dominance in human adult. The Journal of General Psychology.

[30] Baker DH, Graf EW (2009). Natural images dominate in binocular rivalry. Proc Natl Acad Sci USA.

[31] Levelt WJM (1966). The alternation process in binocular rivalry. The British Psychological Society.

[32] Alais D, Melcher D (2007). Strength and coherence of binocular rivalry depends on shared stimulus complexity. Vision Res.

[33] Yang E, Blake R (2012). Deconstructing continuous flash suppression. J Vis.

[34] Huang PC, Maehara G, May KA, Hess RF (2012). Pattern masking: the importance of remote spatial frequencies and their phase alignment. J Vis.

[35] Maehara G, Huang PC, Hess RF (2009). Importance of phase alignment for interocular suppression. Vision Res.

[36] Levelt, W. J. M. On Binocular Rivalry PhD thesis, (1965).

[37] van der Schaaf A, van Hateren JH (1996). Modelling the power spectra of natural images: statistics and information. Vision Res.

[38] Balboa RM, Grzywacz NM (2003). Power spectra and distribution of contrasts of natural images from different habitats. Vision Res.

[39] Nakayama, K., He, Z. J. & Shimojo, S. In Introduction to cognitive science visual cognition (eds. Kosslyn, S. M. & Osherson, D. N.) 1–70 (MIT Press, 1995).

[40] Melcher D, Kowler E (1999). Shapes, surfaces and saccades. Vision Res.

